# Nanoscale Indentation-Induced Crystal Plasticity in CrCoNi Medium-Entropy Alloys Containing Short-Range Order

**DOI:** 10.3390/ma17235932

**Published:** 2024-12-04

**Authors:** Meijing Ren, Fengbo Han, Xu Zhu, Yue Peng, Yanqing Zu, Peitao Liu, Ailing Feng

**Affiliations:** 1 Institute of Physics & Optoelectronics Technology, Baoji University of Arts and Sciences, Baoji 721016, China; 2 Advanced Materials Research Center, Technology Innovation Institute, Abu Dhabi 9639, United Arab Emirates

**Keywords:** crystal plasticity, medium-entropy alloy, short-range order, dislocation slip, geometrically necessary dislocation

## Abstract

CrCoNi medium-entropy alloys (MEAs), characterised by their high configurational entropies, have become a research hotspot in materials science. Recent studies have indicated that MEAs exhibit short-range order (SRO), which affects their deformation mechanisms. In this study, the micro-mechanisms of SRO within the framework of mesoscale continuum mechanics are mathematically evaluated using an advanced, non-local crystal plasticity constitutive framework. Furthermore, a crystal plasticity model considering the impact of SRO on slip is established. By combining nanoindentation simulations and multi-level grain model tensile simulations, the load–displacement and stress–strain curves demonstrated that the presence of SRO increases the hardness of MEAs. More specifically, considering the distribution of shear strain and geometrically necessary dislocations, the heterogeneity of MEAs increases with an increase in the degree of SRO. This study not only enriches the crystal plasticity theory but also provides references for the microstructure and performance regulation of high-performance multi-level grain structure materials.

## 1. Introduction

High- and medium-entropy alloys (HEAs/MEAs) represent a novel category of solid-solution alloys characterised by high configurational entropies. Initially introduced by Yeh et al. [1] and Cantor et al. [2], these alloys contain multiple principal elements in near-equal atomic percentages. This unique composition results in severe lattice distortion and short-range order (SRO), significantly enhancing their mechanical properties (e.g., strength, hardness, plasticity, resistance to high-temperature softening, irradiation resistance, and wear resistance) [3,4]. These properties render HEAs and MEAs promising candidates for applications in the aerospace and nuclear energy sectors. Recent investigations have shown that the optimal structure of a random solid solution can only be achieved at extremely high temperatures [5]. Therefore, the intricate enthalpic interactions between the various elements give rise to SRO structures of different types and degrees [6,7]. These SRO structures impede dislocation slip, which consequently enhances the strength of the material [8,9].

Preliminary research suggested that the constituent elements in HEAs/MEAs occupy random crystal lattice positions, forming an ideal random solid solution. However, recent experiments and computer simulations have demonstrated that the constituent elements in HEAs/MEAs possess an SRO structure. Using X-ray scattering, neutron scattering, and extended X-ray absorption fine-structure techniques, Zhang et al. [8] measured the local structures of CrCoNi MEAs and revealed that the Cr atoms tended to bond with the Ni and Co atoms. For comparison, Ding et al. [10] performed first-principles calculations of the SRO structures present in CrCoNi alloys, and their results were consistent with the experimental findings of Zhang et al. [8]. Furthermore, Zhang et al. [8] observed SRO structures in CrCoNi MEAs using energy-filtered transmission electron microscopy (TEM). The same group [11] also obtained direct experimental evidence of the SRO structures and their interactions with dislocations in CrCoNi MEAs using TEM, electron diffraction, and high-resolution imaging techniques. Chen et al. [12] obtained direct experimental evidence of SRO in CrCoNi MEAs using similar experimental methods and provided the size distributions of the SRO structures. Furthermore, Zhou et al. [7] experimentally detected SRO structures with an average size of >1 nm in Al_9.5_CrCoNi MEAs and revealed that these structures remained stable during the deformation process. Moreover, Wang et al. [13] discovered that ion irradiation can increase the fraction of SRO in HEAs.

To date, research has indicated that the SRO structure can resist dislocation slip in HEAs/MEAs, thereby influencing the proliferation, slip, and accumulation behaviours of the dislocations, altering the stacking fault energy of the alloy and affecting its twinning behaviour [14]. Ding et al. [10] used first-principles calculations to determine the stacking of CrCoNi MEAs with different degrees of SRO. They revealed that by adjusting the degree of SRO, the stacking fault energy could be regulated over a wide range (i.e., −43–30 mJ/m^2^), thereby adjusting the preferential sequence of twinning and the martensitic phase transformation. In addition, Zhang et al. [8] experimentally measured the stacking fault energy of a high-SRO CrCoNi MEA after ageing at 1000 °C, obtaining a value of 23.33 ± 4.31 mJ/m^2^, which is twice that obtained for water-quenched alloys (8.18 ± 1.43 mJ/m^2^). Evidently, high-temperature ageing treatment promoted the formation of short- and medium-range order in CrCoNi MEAs. Further, the SRO structure rendered the atomic arrangement more regular and stable and increased the energy demand for the formation and movement of lamination faults, resulting in a significant increase in the stacking fault energy. Therefore, the layer fault energy of the age-treated alloy was higher than that of the water-quenched alloy, which is directly related to SRO. Furthermore, Zhou et al. [7] used molecular dynamics to study the influence of SRO on the dislocation slip in CrCoNi MEAs during deformation, revealing that SRO inhibited dislocation activation and affected the selection of dislocation slip paths. Moreover, through a quantitative analysis of SRO and mechanical property testing, Zhang et al. [11] demonstrated that the presence of SRO increases the yield strengths of CrCoNi MEAs by ~25%, in addition to doubling the initial work-hardening rate. Chen et al. [12] analysed the interactions between the dislocation cores and SRO and suggested that the presence of SRO leads to tortuous and delayed dislocation motion, thereby enhancing the strain-hardening capability of the alloy [15]. These studies indicate that SRO influences the deformation mechanisms of HEAs/MEAs, ultimately affecting their macroscopic mechanical properties. Consequently, SRO is an important factor to consider in toughening HEAs/MEAs.

Previous studies on HEAs/MEAs have applied the crystal plastic model; Diao et al. [16] used crystal plasticity finite element analysis to investigate the load distribution between the constituent phases in Al_0.3_CoCrFeNi HEAs. In addition, Gao et al. [17] employed a Taylor mean field homogenisation multiscale crystal plasticity model to describe the quasi-static and dynamic behaviours of NiCoCrFe HEAs, whereas Lu et al. [18] used crystal plasticity finite element analysis to examine the relationship between the microstructural evolution and serration behaviour during the cyclic deformation of Fe_49.5_Mn_30_Co_10_Cr_10_C_0.5_ HEAs. However, the crystal plasticity constitutive models employed in these studies did not consider SRO or its influence on the slip mechanisms. Moreover, these models inadequately account for various critical factors such as grain size, grain distribution, and crystal orientation [19,20].

Thus, in this study, a non-local crystal plasticity constitutive model is employed within the continuum mechanics framework, allowing the incorporation of SRO into MEA materials to study its influence on the dislocation slip. Because of the variations during thermal-mechanical processing, the degree of SRO within the material fluctuates. Considering this phenomenon, the influence of distinct degrees of SRO on the evolution of the deformation resistance within slip systems is examined. This study is expected to contribute to unveiling the microstructure-related mechanical properties of HEAs/MEAs and provide a theoretical framework for understanding the toughening mechanisms in such alloys.

## 2. Materials and Methods

### 2.1. Non-Local Crystal Plasticity Constitutive Model

The deformation of a crystal involves elastic deformation induced by lattice distortion and plastic deformation, which are caused by mechanisms such as dislocation slip, twinning, and phase transformation. The total deformation gradient, *F*, is expressed by Equation (1).

(1) $$F=F^{e}F^{P}$$

In addition, the total velocity gradient, *L*, which can be decomposed into the elastic velocity gradient tensor (*L^e^*) and the plastic velocity gradient tensor (*L^p^*), is represented by Equation (2).

(2) $$L=FF^{-1}=L^{e}+L^{P}$$

The slip systems in crystalline materials are determined by their crystal lattices. At ~25 °C, the plastic deformation behaviours of face-centred cubic (FCC) materials, such as CrCoNi MEAs, are primarily induced by dislocation slip. According to the conventional flow rule for metal crystals proposed by Rice [21], *L^p^* can be calculated by Equation (3).

(3) $$L^{P}=\sum_{\alpha =1}^{N}\dot{\gamma^{\alpha }}m^{\alpha }⊗n^{\alpha }$$

where ${\dot{\gamma }}^{\alpha }$ is the slip shear rate on the α^th^ slip system,$\;m^{\alpha }$ denotes the slip direction of the α^th^ slip system, $n^{\alpha }$ is the normal direction of the slip plane, and *N* represents the total number of slip systems. In CrCoNi MEAs, the glide direction is <110>, and the glide plane is {111}; thus, a total of 12 <110> {111} slip systems exist [22].

Using the crystal plasticity finite element framework and methodology based on the work of Peirce et al. [23] in accordance with Schmid’s law [24], the power–law relationship between the shear strain rate (${\dot{\gamma }}^{\alpha }$) generated by the dislocation motion on each slip system, the resolved shear stress ($\tau^{\alpha }$) acting on the slip system and the deformation resistance ($g^{\alpha }$) that must be overcome for dislocation motion, is satisfied by Equation (4).

(4) $${\dot{\gamma }}^{\alpha }={\dot{\gamma }}_{0}^{\alpha }{\left|\frac{\tau^{\alpha }}{g^{\alpha }}\right|}^{n}sgn\left(\tau^{\alpha }\right)$$

where ${\dot{\gamma }}_{0}^{\alpha }$ represents the reference shear strain rate, $\tau^{\alpha }$ is the shear stress, $g^{\alpha }$ is the critical shear stress, and *n* represents the sensitivity index.

The evolution of the deformation resistance in slip systems is controlled by two types of dislocations, namely the stored and geometrically necessary dislocations (GNDs). The strain hardening induced by the accumulation of dislocations is characterised by the evolution of the yield strength variation rate, as outlined in Equation (5).

(5) $${\dot{g}}^{\alpha }=\sum_{\beta }h_{\alpha \beta }{\dot{\gamma }}^{\beta }+\frac{k_{0}{\hat{\alpha }}^{2}G^{2}b}{2\left(g^{\alpha }-g_{0}^{\alpha }\right)}\sum_{\beta }\lambda^{\beta }{\dot{\gamma }}^{\beta }$$

The first term in Equation (5) corresponds to the slip resistance caused by stored dislocations, where the hardening model proposed by Peirce et al. [23] is adopted. In this model, $h_{\alpha \beta }$ represents the latent-hardening modulus, which satisfies Equation (6).

(6) $$h_{\alpha \beta }=qh_{\alpha \alpha }$$

where $h_{\alpha \beta }$ represents the influence of the dislocation slip on its own resistance and the resistances of other slip systems, respectively. Furthermore, $h_{\alpha \alpha }$ can be described by Equation (7).

(7) $$h_{\alpha \alpha }=h_{0}\sec hh^{2}\left|\frac{h_{0}\gamma }{g_{s}-g_{0}}\right|$$

where $h_{0}$ represents the initial hardening modulus, $g_{0}$ denotes the initial value of the slip resistance, $g_{s}$ is the saturation yield stress, and γ is the Taylor cumulative slip of all activated slip systems. The second term in Equation (5) represents the influence of GNDs on plastic deformation hardening, as proposed by Acharya and Beaudoin [25]. In this term,$\;k_{0}$ is a dimensionless material constant, *b* is the Burgers vector, *G* is the elastic shear modulus,$\;{\hat{\alpha }}^{2}$ is a dimensionless constant with a value of 1/3, and $\lambda^{\beta }$ is a measure of the linear dislocation density along the slip plane, which can be expressed by Equation (8).

(8) $$\lambda^{\beta }=\sqrt{\Lambda n^{\beta }\;:\Lambda n^{\beta }}$$

where $n^{\beta }$ represents the normal to the slip plane and Λ denotes the incompatible tensor, which can be expressed using the curl of the plastic deformation gradient tensor, $F^{P}$.

### 2.2. Model Setup

**Figure 1:**
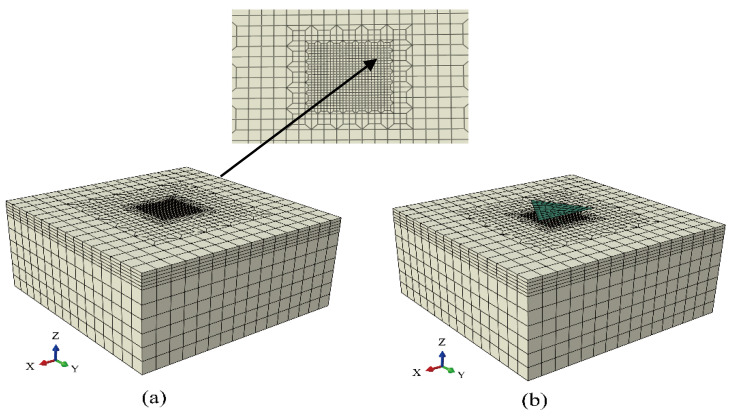
Finite element model of nanoindentation showing (**a**) the specimen (an enlargement of the centre position is shown in the inset) and (**b**) the specimen with the Berkovich indenter.

A crystal plasticity constitutive model was implemented in the user material subroutine (UMAT) within the implicit finite element programme ABAQUS/Standard (2021, Dassault Systemes Simulia Crop., Johnston, RI, USA). This subroutine can capture the stress state and solution-dependent state variables during deformation and provides the material Jacobian matrices required by ABAQUS/Standard. In addition, a three-dimensional nanoindentation model was constructed. Figure 1a shows the specimen mesh, while Figure 1b shows the mesh of the specimen surface assembled with a Berkovich indenter. The Berkovich indenter was set as a discrete rigid body, and the specimen was designated as a deformable component composed of 14,520 eight-node linear solid elements and 16,236 nodes (C3D8). To enhance the accuracy of the finite element simulation, the transition mesh technique was employed to refine the grid in the contact area compared to other regions (minimum element size = 40 nm). In addition, grid-size optimisation was conducted to mitigate excessive element distortion. The dimensions of the specimens were 5.12 × 5.12 × 2.56 μm^3^ (x × y × z). Since the maximum indentation depth did not exceed 70 nm, the thickness of the specimen was sufficiently large to avoid the influence of the substrate. Considering realistic indentation conditions, the bottom and four sides of the specimen model were constrained along three axes to ensure that the specimen adhered to the platform during indentation, leaving the top surface free. The contact surfaces were assumed to be frictionless. During the nanoindentation simulation, the displacement of the Berkovich indenter was controlled at a maximum of 70 nm. The specimen was loaded at a constant speed for 5 s (equivalent to a displacement rate of 14 nm/s), maintained at the maximum displacement for 2 s, and then unloaded at a constant speed for 5 s.

### 2.3. Material Parameters

During the pre-processing step of the finite element simulation, material properties must be assigned to the model elements to provide material-related parameters to the UMAT subroutine. The primary parameters are the elastic constants *C*_11_, *C*_12_, and *C*_44_ [26] of the entropy alloy in CrCoNi, whereas the parameters related to the hardening modulus include the initial hardening modulus, $h_{0}$, the initial value of slip resistance, $g_{0}$, and the saturation yield stress, $g_{s}$. Using the nanoindentation model mentioned in Section 2.2, simulations were conducted to obtain the numerical values of these three parameters from the load–displacement curve. The Burgers vector, *b* [26], shear modulus, *G* [26], rate sensitivity index, *n*, reference strain rate, ${\dot{\gamma }}_{0}$, and dimensionless material constant, $k_{0}$ were obtained through repeated experiments, and the specific values of the relevant parameters are listed in Table 1 and Table 2.

### 2.4. Model Without SRO

**Figure 2:**
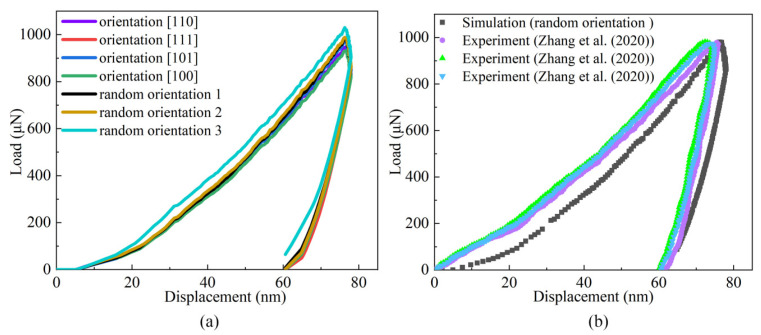
(**a**) Load–displacement curves obtained from the simulation results under seven different orientations. (**b**) Load–displacement curves comparing the simulation results with experimental data from the literature [11] for the CrCoNi MEA.

The load–displacement curves obtained from nanoindentation simulations in ABAQUS are shown in Figure 2. Figure 2a presents the simulation results using different crystal orientations: [110], [111], [101], and [100], along with three random orientations. These curves indicate that crystal orientation has a certain impact on the mechanical properties of the material. The random grain orientations were further characterised and visualised using a pole figure, shown separately in Figure S1.

Figure 2b compares the simulation results for a random orientation with experimental data from [11]. In their study, the exact grain orientation at the indentation site was not determined, which explains why the simulation curves in Figure 2b are based on random orientations. Variations in the experimental curves are attributed to local microstructural differences at different indentation sites, highlighting the material’s heterogeneity. Despite these variations, the comparison between the experimental and simulation curves shows a strong agreement, validating the model’s accuracy.

To quantify this agreement, the area under the load–displacement curves was calculated using the trapezoidal rule in MATLAB (R2016b). The results show a difference of approximately 3.78%, which is within the acceptable threshold of 5%. This minor discrepancy is likely due to local microstructural effects, simplified boundary conditions in the simulation, and statistical sampling limitations. These results confirm that the model accurately captures the mechanical behaviour observed in experiments.

The analysis of yield values included determining the initial hardening modulus, initial slip resistance, and saturation yield stress. The initial hardening modulus was extracted from the initial linear segment of the curve, while the initial slip resistance corresponded to the starting point, representing the material’s initial contact force. The saturation yield stress was derived from the stable portion of the curve, reflecting the material’s saturated yield state.

### 2.5. SRO in the Crystal Plasticity Framework

In this study, the effects of SRO on the mechanical properties of MEAs were investigated, focusing on the role of dislocations and their influence on the material behaviour. In a previous study, Chen et al. [12] applied various techniques to determine the distribution and scale of SRO in FCC VCoNi MEAs at three different temperatures. In addition, Xu et al. [27] used the representative volume element model to conduct a comprehensive simulation analysis of the uniaxial tension and cyclic loading on Cantor-type MEAs at three typical temperatures. They revealed that in CrCoNi MEAs, the degree of SRO varies with the processing temperature, resulting in significant differences in the size distribution of SRO. These SRO structures enhance the slip resistance of a material by impeding dislocation movement. Based on these results, SRO is treated as a nanoscale particle obstacle that affects dislocation slip, ignoring its internal structure and describing it with an average size distribution within a certain range. Its influence on the deformation mechanism is reflected by its influence on the slip system resistance, dislocation density evolution, and stacking fault energy. Specifically, SRO can be described by regulating the initial hardening modulus, $h_{0}$, initial slip resistance, $g_{0}$, and saturated yield stress, $g_{s}$. Positive perturbation terms (ω, ε, and μ) may be introduced to adjust the existing model parameters. The ranges of these disturbance terms can be determined from fitting the stress–strain curve to the tensile test results discussed later. More specifically, ω, ε, and μ are positive and are uniformly distributed within the range of [determined minimum value, determined maximum value]; specific data are given in the following simulation. Hence, the material parameters can be more accurately expressed as follows:

Initial hardening modulus, $h_{0}$, plus the perturbation term, ω; i.e., $h_{0}+\omega$.

Initial slip resistance, $g_{0}$, plus the perturbation term, ε, i.e., $g_{0}+\varepsilon$.

Saturated yield stress, $g_{s}$, plus the perturbation term, μ, i.e., $g_{s}+\mu$.

This approach considers the impact of SRO on the material properties and provides a method to quantify these effects, thereby rendering the model more consistent with the actual behaviour of the material. The specific implementation method uses Python scripts to pre-process the material model and introduce SRO into the nanoindentation model. This allows the indentation simulations to explore the effects of the different degrees of SRO on the material’s hardness, strength, toughness, dislocation movement, and slip behaviour.

**Figure 3:**
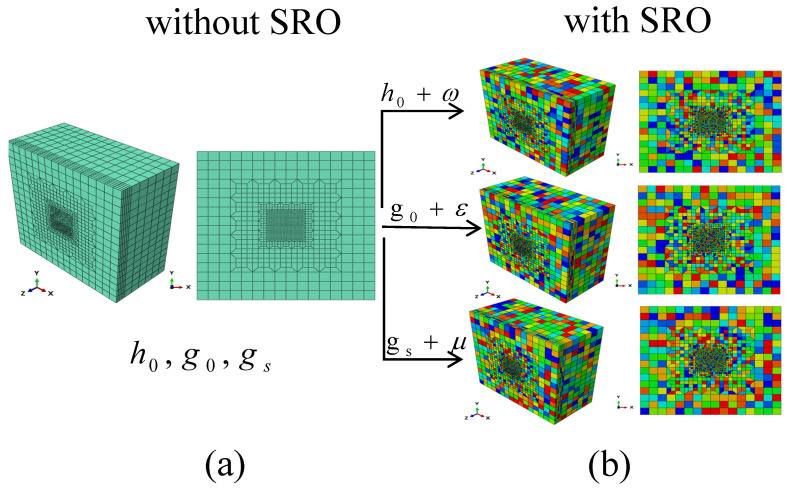
Visualisation of the CrCoNi medium-entropy alloy nanoindentation model incorporating SRO. (**a**) The left column shows the model without SRO, (**b**) the three right columns independently visualise the effects of SRO through perturbations in the Initial hardening modulus, Initial slip resistance, and Saturated yield stress, respectively.

Figure 3 presents the computational framework and simulation results. Figure 3a shows the computational geometry and meshing of the nanoindentation model without SRO, representing a homogeneous material. Figure 3b visualises the heterogeneous distribution of hardness due to varying degrees of SRO. The left column in Figure 3b corresponds to a model without SRO, while the right three columns depict increasing levels of SRO, represented by perturbations in the mechanical properties. These visualisations emphasise how SRO introduces spatial heterogeneity, significantly affecting the mechanical response during nanoindentation.

This modelling approach provides a robust method for the quantification of the effects of SRO on mechanical behaviour, aligning simulation results with experimental observations [11]. The findings underscore the importance of SRO in enhancing hardness and influencing dislocation motion, thereby providing insights into optimising the mechanical performance of CrCoNi MEAs for advanced engineering applications.

## 3. Results and Discussion

### 3.1. Simulation Results: Load–Displacement Curves and Indentation Distributions

**Figure 4:**
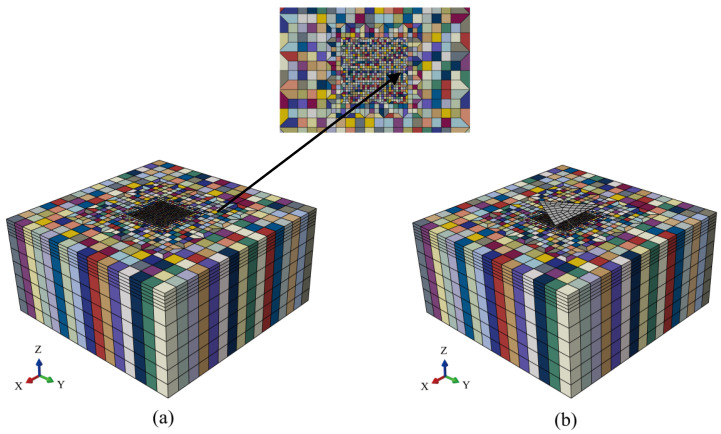
Finite element model of nanoindentation with SRO. (**a**) Morphology of the mesh model without an indenter. The inset shows an enlargement of the centre position. (**b**) Morphology of the mesh model with the Berkovich indenter.

**Figure 5:**
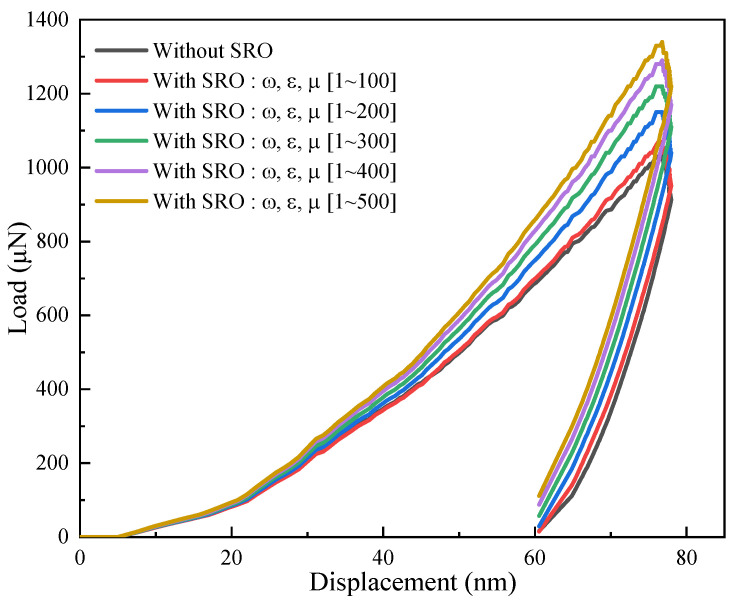
Load–displacement curves obtained from the nanoindentation simulations using the SRO model, in which the parameters ω, ε, and μ are uniformly distributed within the ranges of [1,100], [1,200], [1,300], [1,400], and [1,500]. Higher perturbation factors correlate with an increase in the degree of SRO.

In the model shown in Figure 1, five different degrees of SRO were introduced and simulated during the nanoindentation process. The morphology of the model after the introduction of SRO is shown in Figure 4, where $h_{0}$ represents the initial hardening modulus, $g_{0}$ denotes the initial value of slip resistance and $g_{s}$ signifies the saturated yield stress. These parameters were perturbed within a certain range, i.e., $h_{0}+\omega ,\;g_{0}+\varepsilon ,\;g_{s}+\mu$, wherein the perturbation factors ω, ε, and μ were randomly sampled from the first simulation [1,100], second simulation [1,200], third simulation [1,300], fourth simulation [1,400], and fifth simulation [1,500]. The higher the value range, the greater the degree of SRO. The origins of these five sets of parameters were determined from the tensile simulations using the polycrystalline geometric model described in Section 3.4. The resulting stress–strain curves were then compared with the relevant experimental data (see later) to determine the value ranges for ω, ε, and μ. Therefore, these values can be considered reasonable. All other key parameters remained consistent with those listed in Table 1 and Table 2. Figure 5 shows the load–displacement curves obtained from these five simulations.

**Figure 6:**
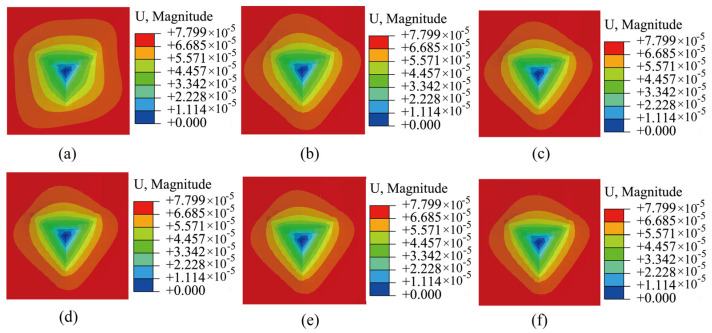
Indentation distribution cloud diagrams with different degrees of SRO: (**a**) without SRO and (**b**–**f**) with increasing degrees of SRO.

The simulation results presented in Figure 5 revealed that as the degree of SRO in the material increases (i.e., the amplitudes of the perturbation factors increase), the slope of the load–displacement curve increases. This corresponds to an increased material hardness, consistent with previously reported experimental results [28,29]. The von Mises stress cloud maps of the nanoindentation displacement under the above six SRO conditions are shown in Figure 6 (side view shown in Figure S2). These contour displacement distribution cloud maps clearly demonstrate that the indentation in the absence of SRO (Figure 6a) is deeper than that in the presence of SRO (Figure 6b–f). Upon increasing the degree of SRO, the indentation gradually diminished, indicating an increase in the material hardness.

### 3.2. Shear Strain Distribution and Statistical Probability Distribution of the Shear Strain

**Figure 7:**
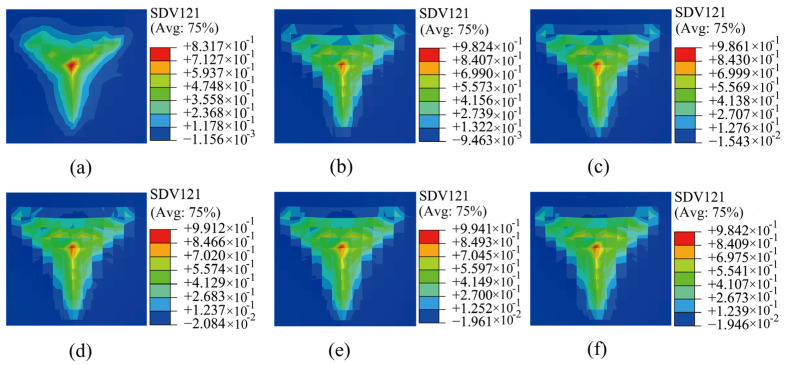
Shear strain distribution diagrams with different degrees of SRO: (**a**) without SRO and (**b**–**f**) with increasing degrees of SRO.

Building on the nanoindentation displacement distribution shown in Figure 6, the material response was further explored by examining the shear strain distribution under the same six conditions. As shown in Figure 7 (side view shown in Figure S3), significant differences are observed between the simulated material results in the presence and absence of SRO. More specifically, in the absence of SRO, the shear strain was relatively continuous and smoothly distributed. However, with the introduction of SRO, the shear strain became discontinuous owing to the irregularity of the local structure, leading to a more discontinuous and uneven distribution of the state variables within the crystal. This demonstrates a stronger microstructural heterogeneity and is consistent with previous observations that the interactions between SRO and the dislocations significantly change the dislocation behaviour and affect the stacking fault energy and local chemical properties, ultimately influencing the material microstructure [30,31]. After introducing SRO, the shear strain became discontinuous, indicating the non-uniformity of the microstructure. This result is consistent with those of recent studies exploring the role of heterogeneity in the lattice friction and back-stress–hardening mechanisms, particularly in the case of multi-principal element alloys. Adjusting the non-uniformity in HEAs/MEAs to promote a strength–ductility synergy is therefore a promising strategy [32].

**Figure 8:**
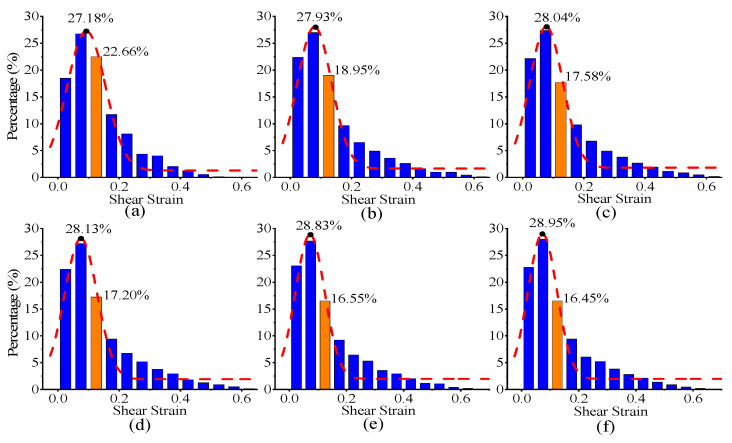
(**a**–**f**) Histograms representing the shear strain distributions in the material models with varying degrees of SRO, corresponding to the values shown in Figure 6a–f. The red dashed lines indicate Gaussian fits of the distributions, while the orange regions highlight the most pronounced changes in the distribution. The black dots mark the peak positions of the distributions, emphasizing the shift in shear strain maxima with increasing SRO.

The maximum indentation moment occurred at 7.056 s; therefore, a statistical analysis of the shear strain distribution was conducted at this time using a total of 3000 nodes that were selected from this region. Figure 8a presents the statistical results for the absence of SRO, whereas Figure 8b–f shows the corresponding results for increasing levels of SRO. In the statistical area displayed in Figure S4, a reduction in the distribution of large strain magnitudes and an increase in the distribution of small strain magnitudes are observed. In particular, the strain distribution probability in the 0.10–0.15 range significantly decreased from 22.656 to 16.446%. Furthermore, the numerical values of the maxima in the Gaussian fitting curve gradually increased from 27.182 to 28.949%. This phenomenon can be attributed to the increased energy barrier for dislocation motion owing to the presence of SRO, which results in restricted dislocation mobility within this range and a subsequent decrease in the dislocation migration rate [30]. Therefore, this enhanced strain distribution reflects the local constraint of dislocation motion by the SRO structures, leading to a localised strain concentration. As the degree of SRO increases, the material may experience a localised stress concentration, resulting in an amplified strain, particularly in regions with higher degrees of order.

### 3.3. Distribution of GNDs

**Figure 9:**
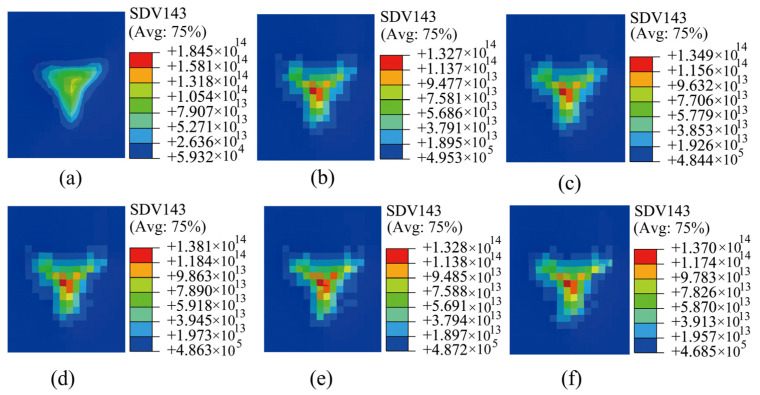
Density distributions of GNDs for simulated indentations with different degrees of SRO: (**a**) without SRO and (**b**–**f**) with increasing degrees of SRO.

The focus of this study was to understand the distribution of GNDs beneath the indentation sites during nanoindentation. These GNDs, which emerge at locations with pronounced strain gradients, significantly influence the material strength and plastic deformation behaviour. Consequently, analysing the density distribution of GNDs beneath the indentation sites yields valuable insights into how SRO affects dislocation formation and distribution. This deeper understanding sheds light on the plastic deformation mechanisms inherent to HEAs/MEAs. Figure 9 presents the simulated maps of GNDs corresponding to the nanoindentation process (side view shown in Figure S5). The pronounced heterogeneity observed in the distribution of GNDs underscores the significant impact of SRO on material behaviour. More specifically, as the degree of SRO increased, the resulting uneven distribution of GNDs indicated an enhanced material hardness and discontinuities in the state variable values, suggesting an increased microstructural heterogeneity.

### 3.4. Polycrystalline Model and Tensile Results

**Figure 10:**
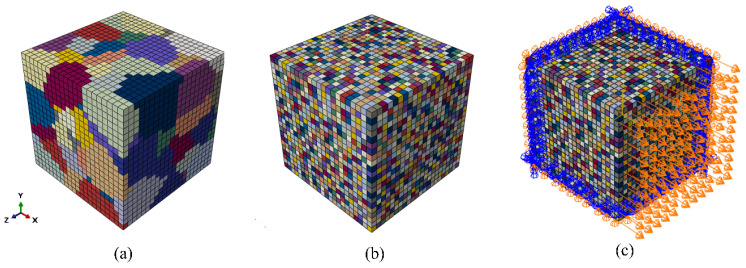
(**a**) Polycrystalline geometric model containing 100 grains with random orientations generated using Neper [32]. (**b**) Representative volume element (RVE) of a polycrystalline cube exhibiting SRO. (**c**) RVE of a polycrystalline cube with SRO subjected to tensile stress, the direction of which is indicated by the orange arrows.

The transition from nanoindentation studies to a polycrystalline geometric model retained the applicability of the non-local crystal plasticity constitutive model and its associated parameters. The polycrystalline geometric model (Figure 10) was created using the Neper program (version 4.3.0) [32], employing C3D8 elements with a unit size of approximately 182 µm and resulting in an overall model size of 4000 × 4000 × 4000 µm^3^. The model comprised 100 grains, each generated as Voronoi polyhedra based on 100 random seed points. Accordingly, the average grain size was approximately 865 µm, which closely matches the grain size observed in the experiments.

**Figure 11:**
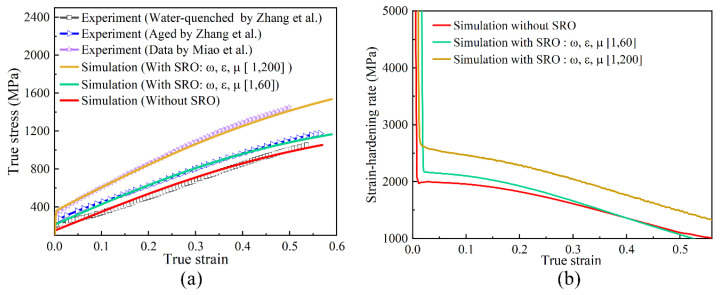
(**a**) True stress–true strain curves comparing the simulation results with the experimental data reported by Zhang et al. [11] and Miao et al. [32]. (**b**) Strain-hardening rate–true-strain curves.

After parameterising the polycrystalline model, a stretching simulation was performed along the *x* direction using the other three fixed faces. The resulting stress–strain and strain hardening rate curves were compared with the corresponding experimental results, demonstrating an excellent match (Figure 11). The obtained strain hardening rate curve indicates that during plastic deformation, the strain hardening rate gradually decreased as the degree of deformation increased. This decrease was significantly slower than that observed during the elastic deformation stage, which is consistent with the experimental results reported by Miao et al. [32].

As shown in Figure 11a, tensile simulations were conducted on a polycrystalline geometric model using parameters largely consistent with those from the nanoindentation model. This ensures continuity between the two modelling approaches. However, specific adjustments were made to the ω, ε and μ values, as shown in Figure 11a, to better represent the influence of SRO in the polycrystalline samples. By comparing the simulation results with the experimental data [31,33], a high degree of consistency was observed, validating the choice of these parameters. This simulation analysis revealed that as the degree of SRO increased, the yield point on the stress–strain curve also increased, leading to enhanced yield strength. This further confirms the influence of SRO on the material properties. Moreover, in the plastic deformation stage (Figure 11b), the strain hardening rate decreased gradually with increasing strain. Therefore, the accuracy and reliability of this model in describing the material behaviour provide a solid foundation for further research and engineering applications. In addition, this model demonstrates versatility across different loading conditions and geometric shapes, rendering it widely applicable.

## 4. Conclusions

An advanced non-local crystal plasticity constitutive framework was employed by integrating SRO effects to shed light on the mechanisms impacting dislocation slip in MEAs. As the degree of SRO in the material increased, the slope of the load–displacement curve increased, requiring a greater force to achieve the same indentation depth. This indicates an increase in the material hardness, which is consistent with the simulation results. In the absence of SRO, the distribution plots of the various state variables appeared smooth and relatively continuous. However, after introducing SRO, the numerical values of the state variables appeared discontinuous, indicating a stronger heterogeneity in the microstructure. In addition, a significant change in the plastic behaviour of the material was observed with an increase in the degree of SRO. This was attributed to the SRO structure increasing the energy barrier for dislocation movement, thereby restricting the dislocation mobility, particularly within the range of 0.10–0.15. This phenomenon resulted in a reduction in regions with large plastic strains and an increase in regions with smaller strains, indicating the direct influence of SRO on the dislocation slip mechanism. Additionally, the indentation depth decreased with an increase in the degree of SRO, further confirming a reduction in the plastic deformation capability of the material. Evidently, SRO is crucial in determining the material properties of MEAs, including the material strain behaviour, defect distribution, and dislocation activity, ultimately influencing the material performance and stability. With this deeper understanding of the role of SRO, material design can be more carefully tuned to meet diverse engineering requirements. Future research should continue to explore the mechanisms underlying the effect of SRO on material properties, expand its application in materials science and engineering, and provide more reliable and controllable methods for material design and manufacturing. Further investigations in this field are expected to provide valuable insights into the development of MEAs with tailored properties. This will facilitate their application in areas such as aerospace components, nuclear reactor materials, and wear-resistant industrial parts, contributing to improved performance and durability in these critical applications.

## Figures and Tables

**Table 1:** Crystal plasticity model parameters for the CrCoNi MEAs.

Slip Systems	${\dot{\gamma }}_{0}$	*n*	$k_{0}$	$h_{0}\;\left(\mathrm{MPa}\right)$	$\tau_{0}\;\left(\mathrm{MPa}\right)$	$\tau_{s}\;\left(\mathrm{MPa}\right)$
Basal<*a*>	0.001	15	28	391	70	624

**Table 2:** Elastic constants of the α-phase for the CrCoNi MEAs.

$C_{11}\;\left(\mathrm{MPa}\right)$	$C_{12}$ (MPa)	$C_{44}\;\left(\mathrm{MPa}\right)$	*b* (mm)	$G\;\;\left(\mathrm{MPa}\right)$
249,400	159,000	1,384,000	2.522 × 10^−6^	89,100

## Data Availability

The original contributions presented in this study are included in the article/Supplementary Materials. Further inquiries can be directed to the corresponding authors.

## Supplementary Materials

The following supporting information can be downloaded at: https://www.mdpi.com/article/10.3390/ma17235932/s1, Figure S1: Inverse pole figure illustrating the random orientations of grains. The blue dots represent the crystallographic orientations of individual grains within the triangular stereographic projection. Figure S2: Displacement cloud side view with different degrees of SRO, where (a) to (f) correspond one-to-one with (a) to (f) in Figure 6. Figure S3: Shear strain distribution diagram side view of different degrees of SRO, where (a) to (f) correspond one-to-one with (a) to (f) in Figure 7. Figure S4: Shear strain distribution statistics selected area (Insert is a side view enlargement). Figure S5: GNDs density distribution diagram side view with different degrees of SRO where (a) to (f) correspond one-to-one with (a) to (f) in Figure 9.
