# Supplementary material for: Nanoscale Indentation-Induced Crystal Plasticity in CrCoNi Medium-Entropy Alloys Containing Short-Range Order
Source: Materials (Basel). 2024 Dec 4;17(23):5932. doi: 10.3390/ma17235932 (PMC11642976; doi:10.3390/ma17235932)
Supplement: Supplementary file 1 [file materials-17-05932-s001.zip › materials-3329565-supplementary.pdf]

Supplementary Material

Nanoscale Indentation-Induced Crystal Plasticity in CrCoNi Medium-Entropy Alloys Containing Short-Range Order

Meijing Ren[a], Fengbo Han[b, *], Xu Zhu[a], Yue Peng[a], Yanqing Zu[a], Peitao Liu[a], Ailing Feng[a, *]

[a] Institute of Physics & Optoelectronics Technology, Baoji University of Arts and Sciences, Baoji, 721016, China
[b] Advanced Materials Research Center, Technology Innovation Institute, Abu Dhabi, 9639, United Arab Emirates

* Corresponding authors
Ailing Feng, email: ailingfeng@bjwlxy.edu.cn
Fengbo Han, email: Fengbo.Han@tii.ae

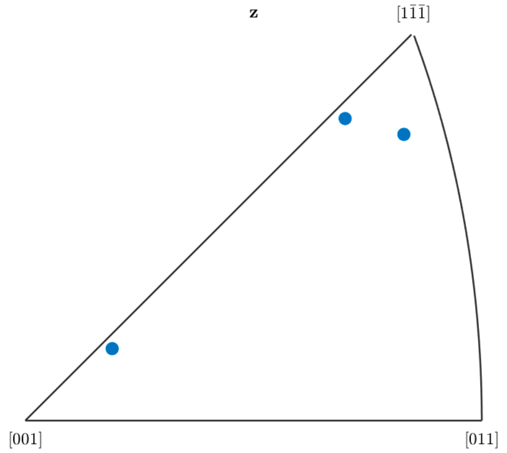

Figure S1. Inverse pole figure illustrating the random orientations of grains. The blue dots represent the crystallographic orientations of individual grains within the triangular stereographic projection.

Inverse pole figure used to represent the random crystal orientations in the nanoindentation simulation. The stereographic triangle illustrates crystallographic directions with [001] at the bottom left, [011] at the bottom right, and $[1\bar{1}\bar{1}]$ at the top. The blue dots indicate specific crystal orientations utilized in the simulation. The crystal orientations are defined by the Euler angles (256.6°, 119.9°, 122.9°), (238.9°, 83.1°, 81.6°), and (47.4°, 133.1°, 52.9°), providing a clear and intuitive visualization of the crystal orientation distribution in the polycrystalline material, which influences its mechanical properties.

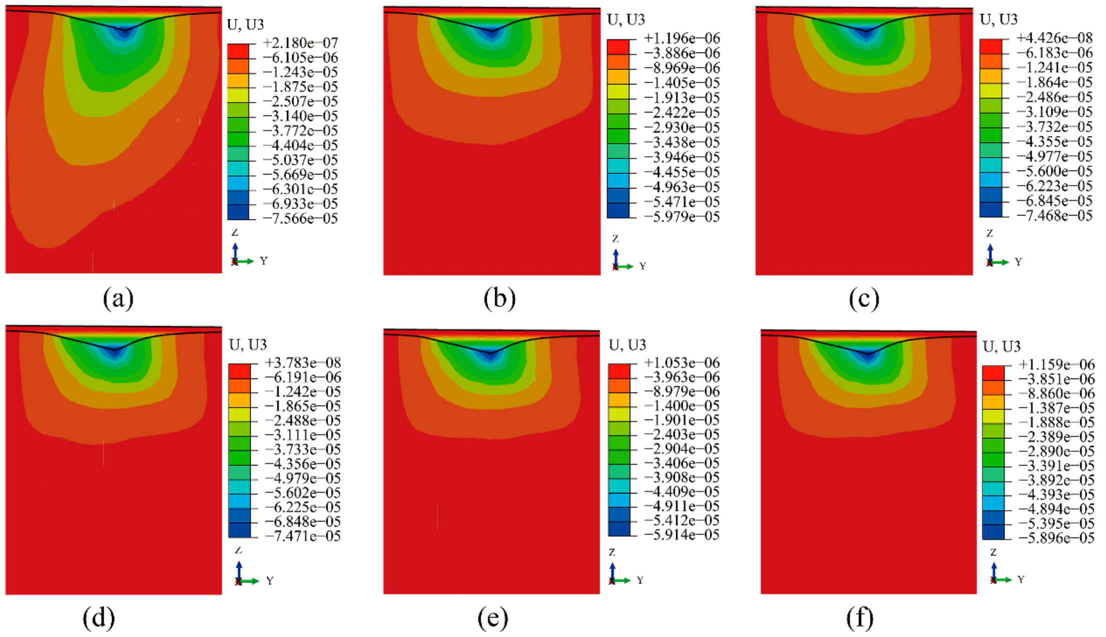

Figure S2. Displacement cloud side view with different degrees of SRO, where (a) to (f) correspond one-to-one with (a) to (f) in Figure 6.

Provides a side view of the displacement cloud diagrams corresponding to different degrees of SRO, aligning with Figures 6(a) to 6(f) in the main text. These views emphasize the progression of displacement distribution as SRO intensity increases. Asymmetry is observed in Figure S2(a), primarily originating from the shape of the Berkovich indenter used in the simulation. Due to the triangular pyramid shape of the

Berkovich indenter, stress concentrations are inherently asymmetric because of slight variations in boundary conditions during the simulation. Additional factors, such as mesh discretization or local heterogeneities in the material, could also contribute to the observed asymmetry.

Reduction of Asymmetry by SRO: The inclusion of SRO introduces nanoscale obstacles that homogenize the deformation process by impeding dislocation movement in a distributed manner. This effect reduces localized stress concentrations and promotes a more uniform stress distribution. SRO acts as a stabilizing feature, mitigating asymmetry in the stress field by redistributing the load more evenly across the microstructure.

Relationship Between Microstructure and Mechanical Behavior: The reduced asymmetry in stress distribution indicates that SRO enhances the material's resistance to localized deformation, resulting in a more uniform load transfer. This directly affects the material's mechanical behavior, as evidenced by the increased hardness and reduced stress heterogeneity observed in the simulations. The interplay between SRO and dislocation dynamics smoothens the stress gradients, thereby reducing asymmetry in both stress and strain fields during indentation.

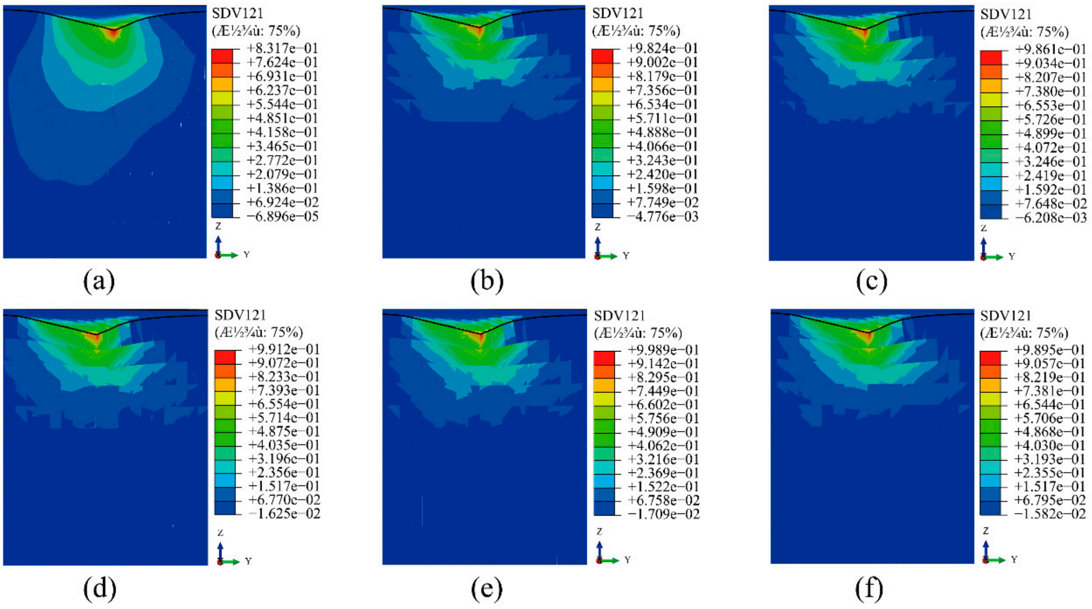

Figure S3. Shear strain distribution diagram side view of different degrees of SRO, where (a) to (f) correspond one-to-one with (a) to (f) in Figure 7.

The contours illustrate variations in shear strain (SDV121) within the material, highlighting areas of high strain concentration in red and yellow, and low strain in blue. The progression from (a) to (f) shows the impact of increasing SRO on the distribution and intensity of shear strain within the specimen.

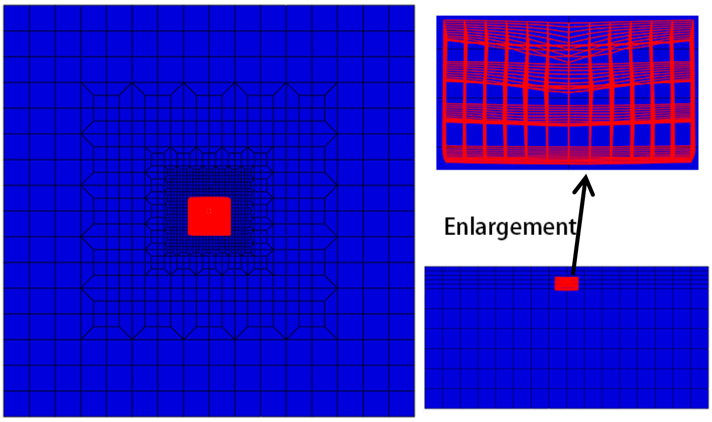

Figure S4. Shear strain distribution statistics selected area (Insert is a side view enlargement).

Shear strain distribution statistics for the selected area, with an insert showing a side view enlargement. The maximum indentation moment occurred at 7.056 seconds; therefore, a statistical analysis of the shear strain distribution at this time was conducted using a total of 3,000 nodes selected from this region. The selected area, located directly below the indenter tip, represents the region with the highest deformation in the model. The side view clearly shows the selected depth, and the enlarged view reveals the detailed mesh structure of the selected nodes. Figure 8(a) presents the statistical results for the absence of SRO, whereas Figures 8(b)–8(f) show the corresponding results for increasing levels of SRO, highlighting variations in shear strain within the selected region.

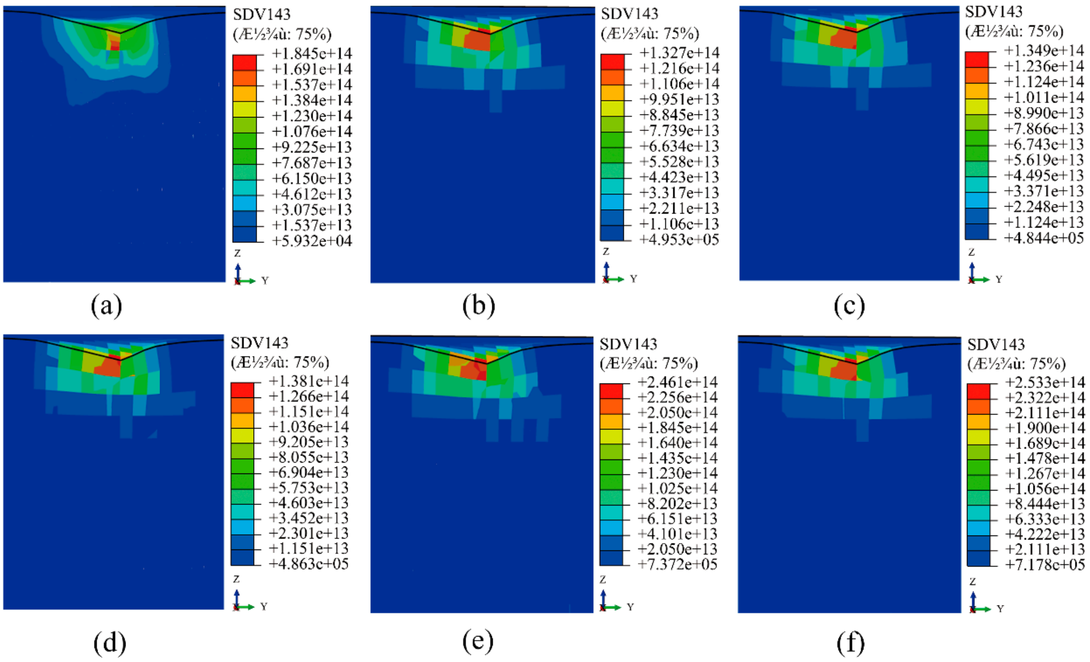

Figure S5. GNDs density distribution diagram side view with different degrees of SRO where (a) to (f) correspond one-to-one with (a) to (f) in Figure 9.

Side view of the GNDs density distribution diagram with varying degrees of SRO, where (a) to (f) correspond one-to-one with Figures 9(a) to 9(f) in the main text. The color-coded diagrams illustrate the distribution of GNDs density, with higher densities highlighted in red and yellow, and lower densities in blue. These diagrams provide a visual representation of how the GNDs density distribution changes with

different degrees of SRO, which is crucial for understanding the material properties and behaviors under varying conditions.
